# Supplementary material for: p97 regulates GluA1 homomeric AMPA receptor formation and plasma membrane expression
Source: Nat Commun. 2019 Sep 9;10:4089. doi: 10.1038/s41467-019-12096-7 (PMC6733861; doi:10.1038/s41467-019-12096-7)
Supplement: Supplementary file 1 — Supplementary information [file 41467_2019_12096_MOESM1_ESM.pdf]

p97 regulates GluA1 homomeric AMPA receptor formation and plasma membrane expression

Ge et al.

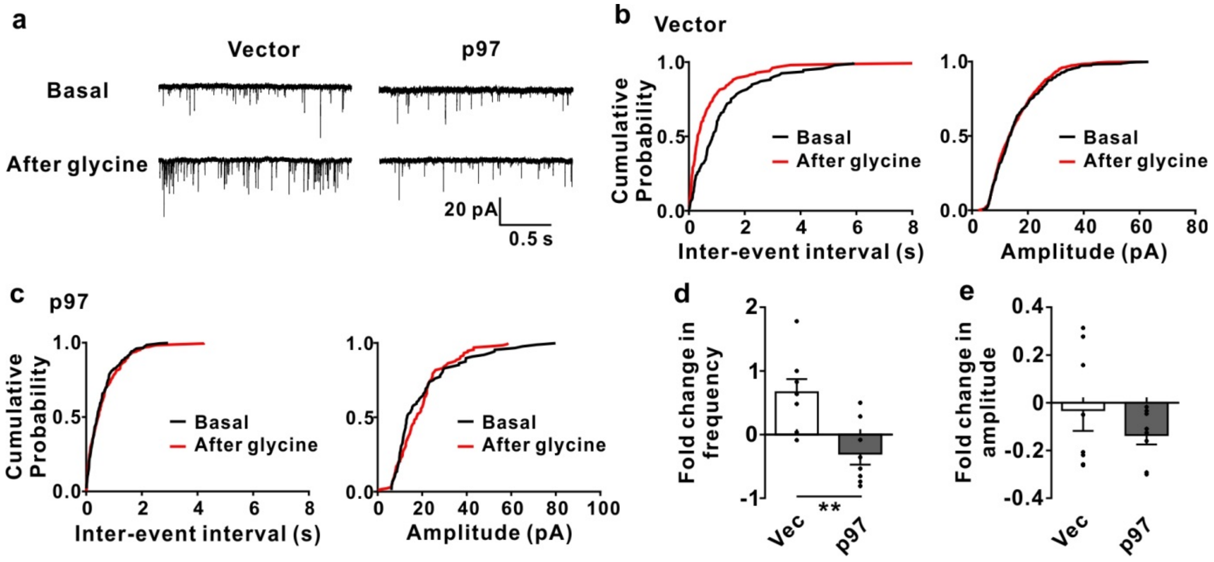

Supplementary Figure 1. Overexpression of p97 occludes glycine-induced chemical LTP in cultured hippocampal neurons

Hippocampal neurons were transfected with Vector or p97, along with GFP to detect the transfected cells for recording. Chemical LTP was induced by bath application of glycine (200 μM; 3 min) 48 hours after transfection. Glycine treatment reliably induced LTP of mEPSCs, as manifested by the significant increase in mEPSC frequency 25 min after glycine stimulation, in control neurons transfected with vector (Vector), but not in p97-overexpressing neurons (p97). From **a-e** are represented traces (**a**), cumulative probability plots of inter-event intervals and amplitudes (**b** and **c**), and averaged bar graphs of the fold changes in frequency (**d**) and amplitude (**e**) of mEPSCs recorded 5 min before and 25 min after glycine application in neurons transfected with Vector (Vec, n=8 cells) or p97 (p97, n=8 cells) (** $p < 0.01$, two-tailed t-test). The error bars represent SEM.

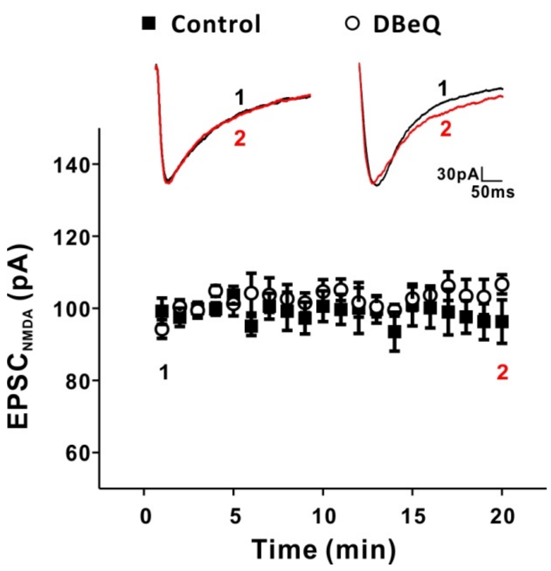

Supplementary Figure 2. Inhibition of p97 does not affect NMDAR-mediated EPSCs in hippocampal slices

NMDAR-mediated excitatory postsynaptic currents (EPSCs) were evoked by electrical stimulation of schaffer collateral inputs at a holding membrane potential of -30mV in Mg^{2+}-free perfusion solution containing AMPAR blocker DNQX (10 μM). Intracellular application of p97 inhibitor DBeQ (11μM) through the recording pipette didn't change NMDAR-mediated EPSCs (Control: n=7 cells; DBeQ: n=8 cells). The error bars represent SEM.

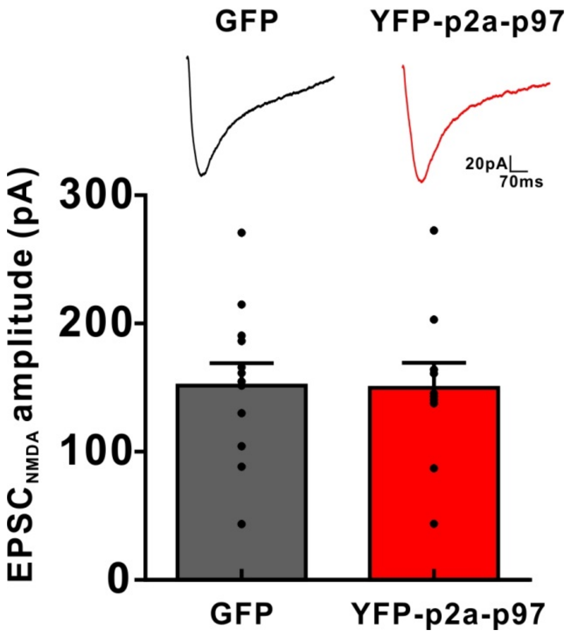

Supplementary Figure 3. Overexpression of p97 does not change NMDAR mediated EPSCs in hippocampal slices

AAV viral vector containing GFP or YFP-p2a-p97 was injected into the ventricles of mice at P0. Evoked EPSCs were recorded in visually identified GFP or YFP positive CA1 pyramidal neurons in hippocampal slices prepared at P14-21. NMDAR-mediated postsynaptic currents were evoked by electrical stimulation of schaffer collateral inputs at holding membrane potential of -30mV in Mg^{2+}-free perfusion solution containing AMPAR blocker DNQX (10 μM). Overexpression of p97 didn't change NMDAR mediated EPSCs (GFP: n=14 cells; YFP-p2a-p97: n=10 cells). The error bars represent SEM.
