# Supplementary material for: p97 regulates GluA1 homomeric AMPA receptor formation and plasma membrane expression
Source: Nat Commun. 2019 Sep 9;10:4089. doi: 10.1038/s41467-019-12096-7 (PMC6733861; doi:10.1038/s41467-019-12096-7)
Supplement: Supplementary file 3 — Reporting Summary [file 41467_2019_12096_MOESM3_ESM.pdf]

nature research

Corresponding author(s): Yu Tian Wang, Yuan Ge, Jie Lu

Last updated by author(s): Aug 18, 2019

Reporting Summary

Nature Research wishes to improve the reproducibility of the work that we publish. This form provides structure for consistency and transparency in reporting. For further information on Nature Research policies, see Authors & Referees and the Editorial Policy Checklist.

Statistics

For all statistical analyses, confirm that the following items are present in the figure legend, table legend, main text, or Methods section.

n/a	Confirmed	
☐	☒	The exact sample size (*n*) for each experimental group/condition, given as a discrete number and unit of measurement
☐	☒	A statement on whether measurements were taken from distinct samples or whether the same sample was measured repeatedly
☐	☒	The statistical test(s) used AND whether they are one- or two-sided<br>*Only common tests should be described solely by name; describe more complex techniques in the Methods section.*
☒	☐	A description of all covariates tested
☐	☒	A description of any assumptions or corrections, such as tests of normality and adjustment for multiple comparisons
☐	☒	A full description of the statistical parameters including central tendency (e.g. means) or other basic estimates (e.g. regression coefficient) AND variation (e.g. standard deviation) or associated estimates of uncertainty (e.g. confidence intervals)
☒	☐	For null hypothesis testing, the test statistic (e.g. F, t, r) with confidence intervals, effect sizes, degrees of freedom and P value noted<br>*Give P values as exact values whenever suitable.*
☒	☐	For Bayesian analysis, information on the choice of priors and Markov chain Monte Carlo settings
☒	☐	For hierarchical and complex designs, identification of the appropriate level for tests and full reporting of outcomes
☒	☐	Estimates of effect sizes (e.g. Cohen's *d*, Pearson's *r*), indicating how they were calculated

Our web collection on statistics for biologists contains articles on many of the points above.

Software and code

Policy information about availability of computer code

Data collection	Quantity One (Western blot), Image Lab (Western blot), MetaMorph (immunostaining), Pclamp 10 (Electrophysiology)
Data analysis	Image J (Western blot and immunostaining), Image Lab (Western blot), MASCOT (Mass spec), Mini Analysis (mEPSC), Pclamp 10 (Electrophysiology)

For manuscripts utilizing custom algorithms or software that are central to the research but not yet described in published literature, software must be made available to editors/reviewers. We strongly encourage code deposition in a community repository (e.g. GitHub). See the Nature Research guidelines for submitting code & software for further information.

Data

Policy information about availability of data

All manuscripts must include a data availability statement. This statement should provide the following information, where applicable:

- Accession codes, unique identifiers, or web links for publicly available datasets
- A list of figures that have associated raw data
- A description of any restrictions on data availability

All the original blots images are provided as the Source Data file. The data that support the findings of this study are available from the corresponding author upon reasonable request.

Field-specific reporting

Please select the one below that is the best fit for your research. If you are not sure, read the appropriate sections before making your selection.

☒ Life sciences ☐ Behavioural & social sciences ☐ Ecological, evolutionary & environmental sciences

For a reference copy of the document with all sections, see nature.com/documents/nr-reporting-summary-flat.pdf

Life sciences study design

All studies must disclose on these points even when the disclosure is negative.

Sample size	No sample size calculation was performed. The sample size were chosen based on the previous publications (Man et al., Neuron, 2003; Liu et al., Science, 2004; Ahmadian et al., EMBO J, 2004).
Data exclusions	No data were excluded from data analysis.
Replication	All experiments were repeated at least in 2 independent experiments. All attempts at replication were successful.
Randomization	For cell transfection, cell lines or cultured neurons were randomly assigned to experimental groups. For immunofluorescent imaging, random transfected COS7 cells or cultured hippocampal neurons were selected. For AAV injection, new born mice were randomly assigned to AAV-GFP or AAV-YFP-p2a-Clptm1 injection groups. For electrophysiology, random transfected HEK293 cells, cultured hippocampal neurons, or CA1 pyramidal neurons were selected for recordings.
Blinding	The investigators were blinded to group allocation during data collection and analysis.

Reporting for specific materials, systems and methods

We require information from authors about some types of materials, experimental systems and methods used in many studies. Here, indicate whether each material, system or method listed is relevant to your study. If you are not sure if a list item applies to your research, read the appropriate section before selecting a response.

Materials & experimental systems

n/a	Involved in the study	
☐	☒	Antibodies
☐	☒	Eukaryotic cell lines
☒	☐	Palaeontology
☐	☒	Animals and other organisms
☒	☐	Human research participants
☒	☐	Clinical data

Methods

n/a	Involved in the study	
☒	☐	ChIP-seq
☒	☐	Flow cytometry
☒	☐	MRI-based neuroimaging

Antibodies

Antibodies used	anti-GluA1 and anti-GluA2 were raised in the lab. anti-HA (Roche, Cat#11867431001), anti-p97 (Fitzgerald, Cat#10R-P104A), anti-GFP (Invitrogen, Cat#11122), anti-Na+K+ATPase antibody (Abcam, Cat#ab7671), anti-b-Actin antibody (Sigma, Cat#AC74), anti-GluA1 (Calbiochem, Cat#PC246), GluA2 (Millipore, Cat#MAP397), anti-vGluT1 (Synaptic Systems, Cat#135304), anti-MAP2 (Abcam, Cat#ab5392).
Validation	lab-raised anti-GluA1 and anti-GluA2 antibodies were validated in Fig 1a. All the other antibodies used are commercially available and validated.

Eukaryotic cell lines

Policy information about cell lines

Cell line source(s)	COS7 (ATCC, Cat#CRL-1651), HEK293 (ATCC, Cat#CRL-1573)
Authentication	The cell lines used were not authenticated.
Mycoplasma contamination	The cell lines were not tested for mycoplasma contamination.
Commonly misidentified lines (See ICLAC register)	None

Animals and other organisms

Policy information about studies involving animals; ARRIVE guidelines recommended for reporting animal research

Laboratory animals	Sprague-Dawley rats (For neuron culture, male and female, embryonic day 18; for electrophysiological recordings, male, 2-4 weeks old), C57BL/6 mice (male; AAV injection at postnatal day 0; and electrophysiological recordings at 2-3 weeks old)
Wild animals	No wild animal were used.
Field-collected samples	No field-collected samples were used.
Ethics oversight	All experimental procedures with animals were conducted following the guidelines of the Canadian Council for Animal Care and approved by the University of British Columbia Animal Care Committee.

Note that full information on the approval of the study protocol must also be provided in the manuscript.
