# Supplementary material for: p97 regulates GluA1 homomeric AMPA receptor formation and plasma membrane expression
Source: Nat Commun. 2019 Sep 9;10:4089. doi: 10.1038/s41467-019-12096-7 (PMC6733861; doi:10.1038/s41467-019-12096-7)
Supplement: Supplementary file 4 — Source Data [file 41467_2019_12096_MOESM4_ESM.pdf]

Fig 1a

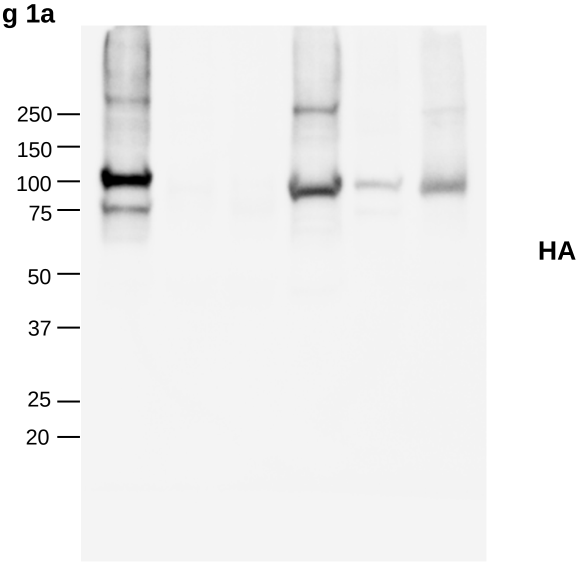
250
150
100
75
50
37
25
20
HA

Fig 1c

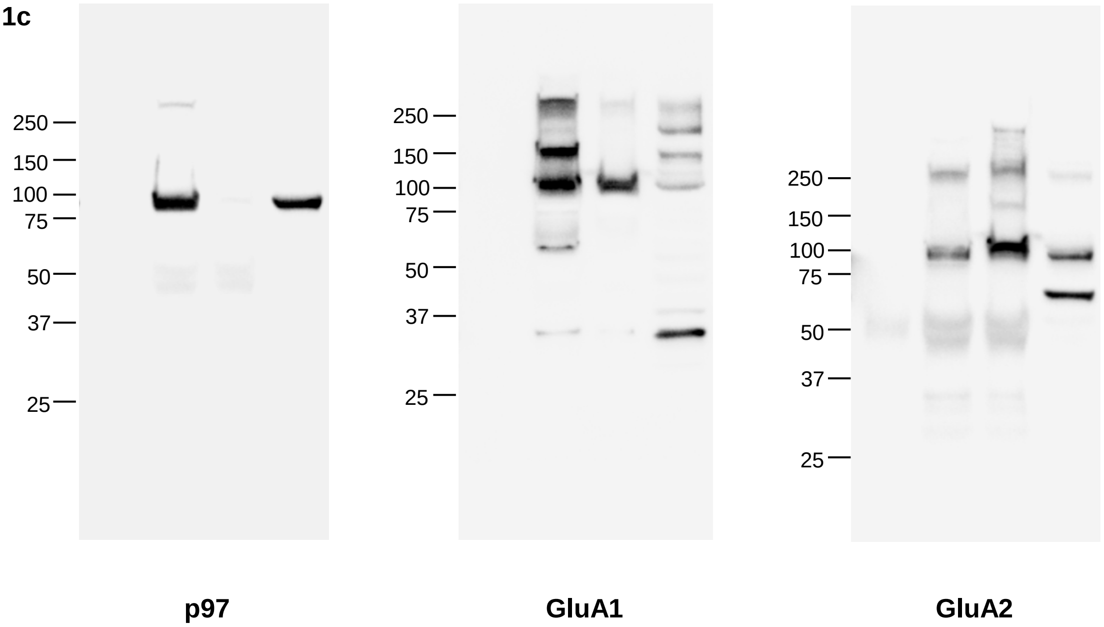
250
150
100
75
50
37
25
p97
GluA1
GluA2

Fig 1d

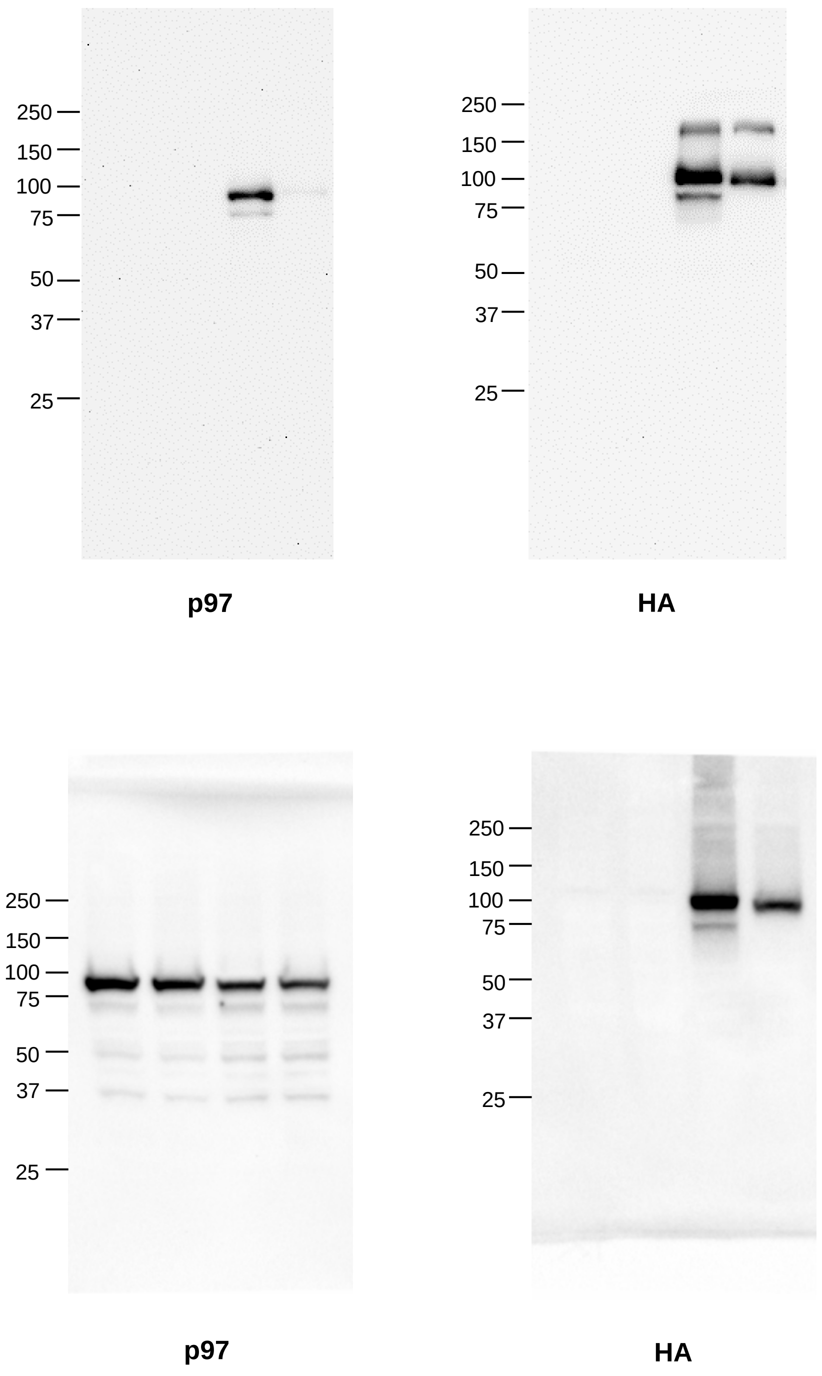
250
150
100
75
50
37
25
p97
250
150
100
75
50
37
25
HA
250
150
100
75
50
37
25
p97
250
150
100
75
50
37
25
HA

Fig 1e

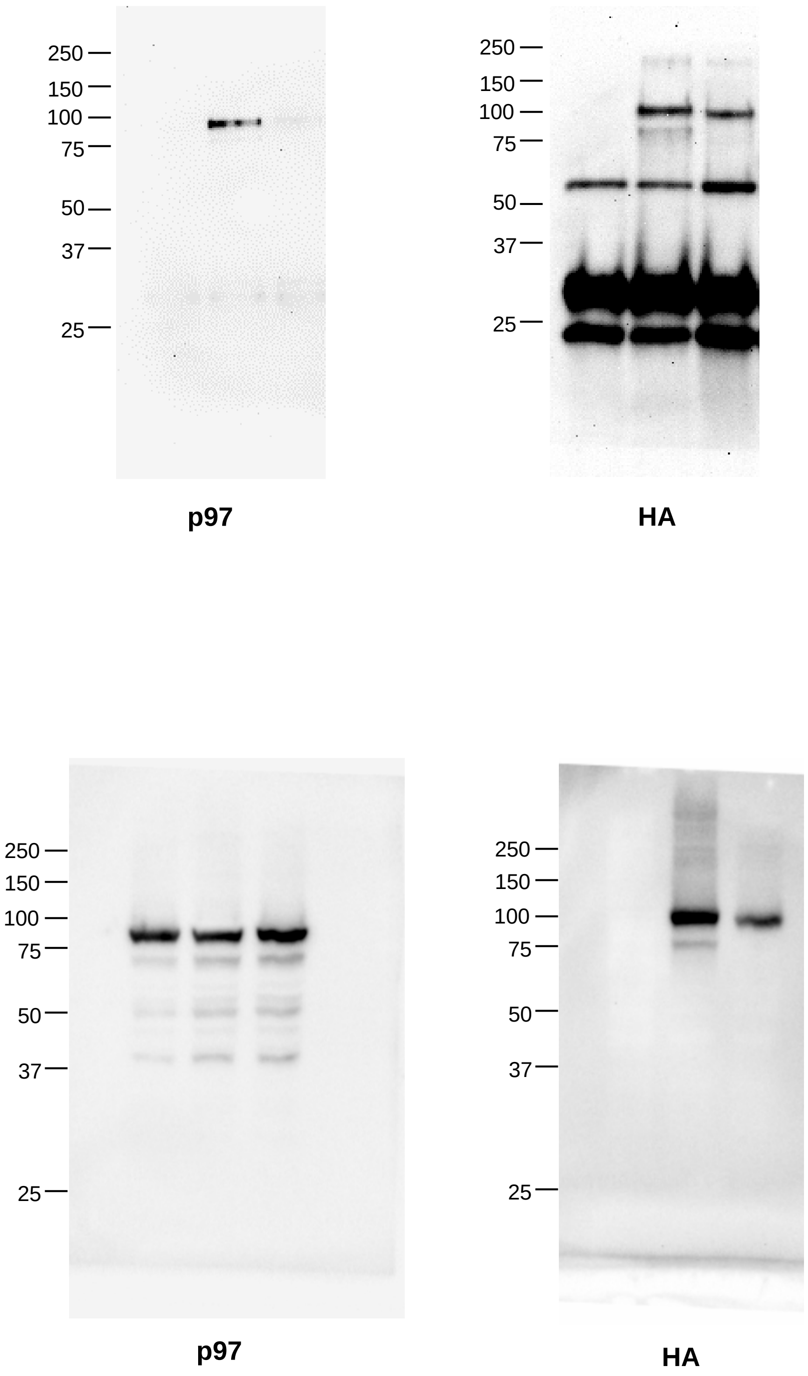

Fig 1f

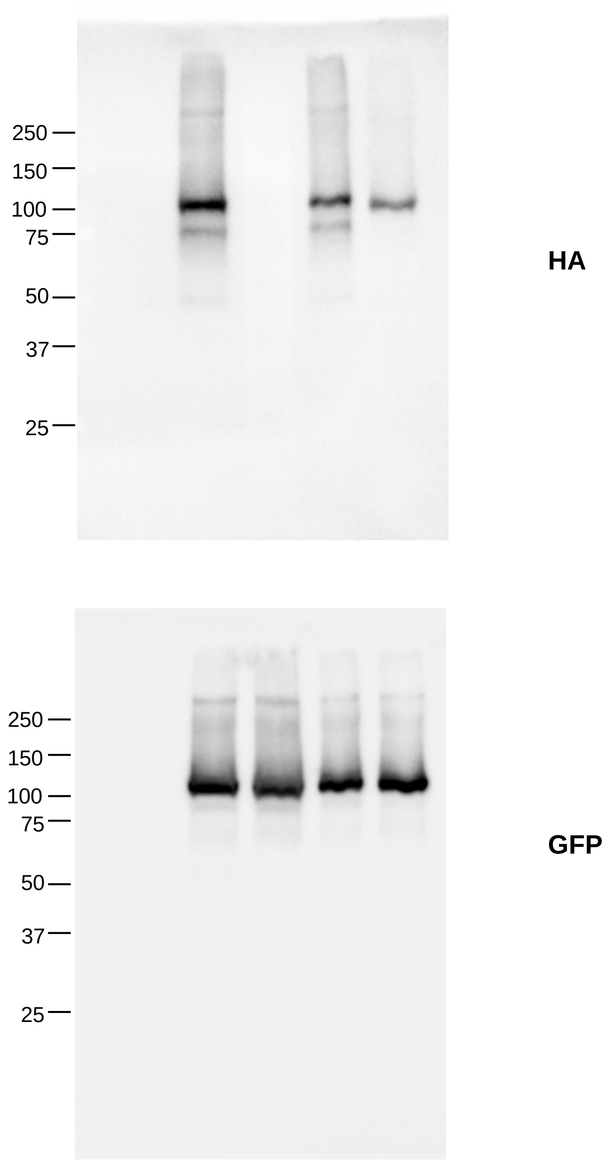

Fig 1h

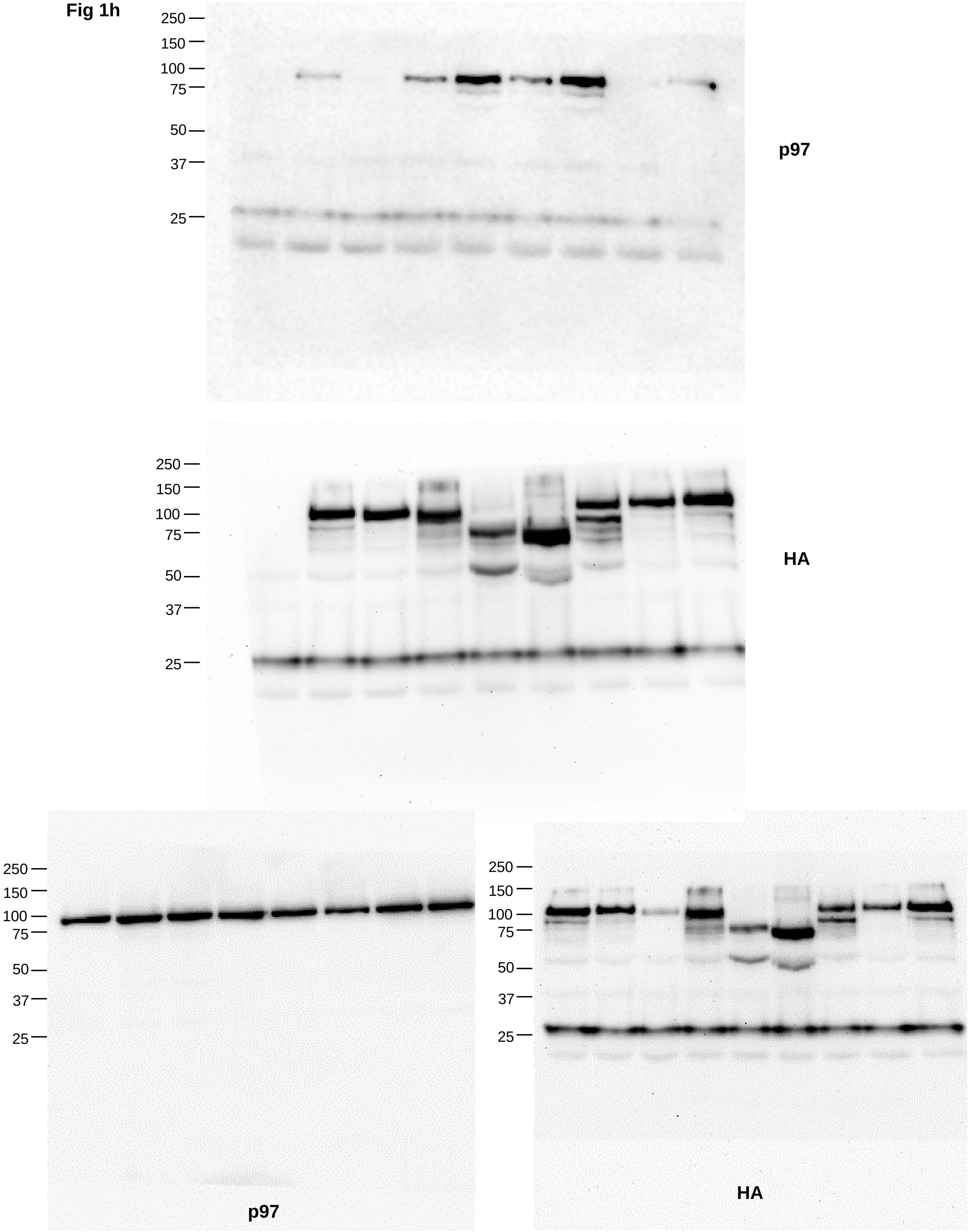
250
150
100
75
50
37
25
p97
250
150
100
75
50
37
25
HA
250
150
100
75
50
37
25
p97
250
150
100
75
50
37
25
HA

Fig 1i

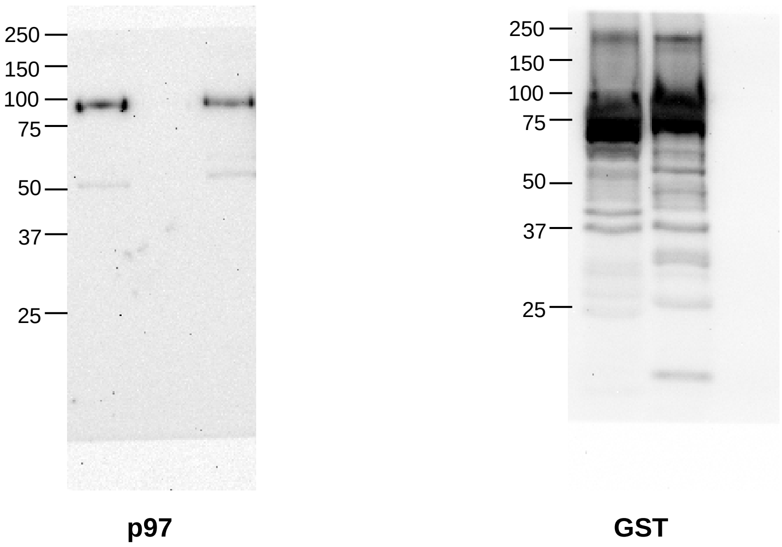

Fig 1j

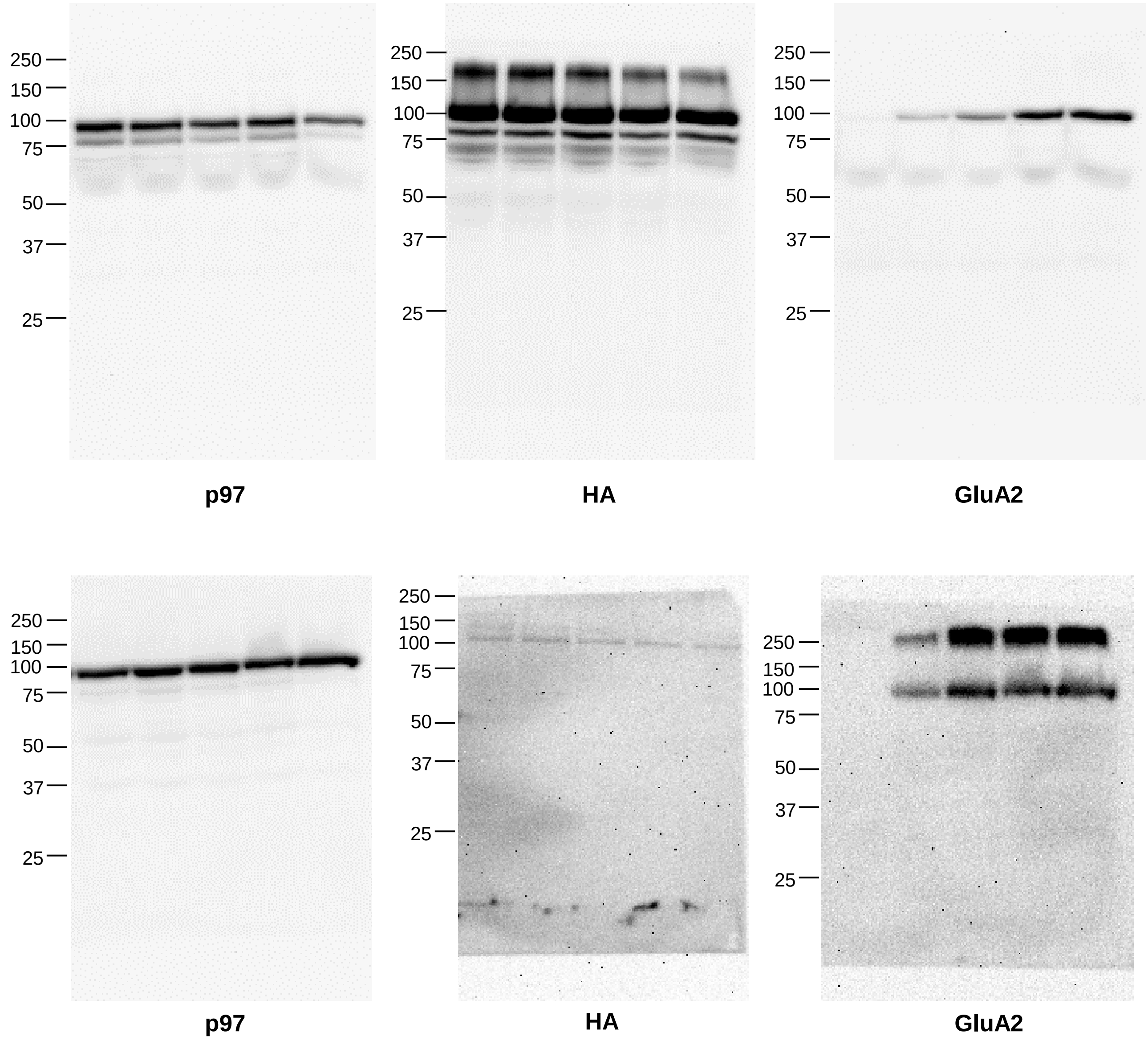

Fig 1k

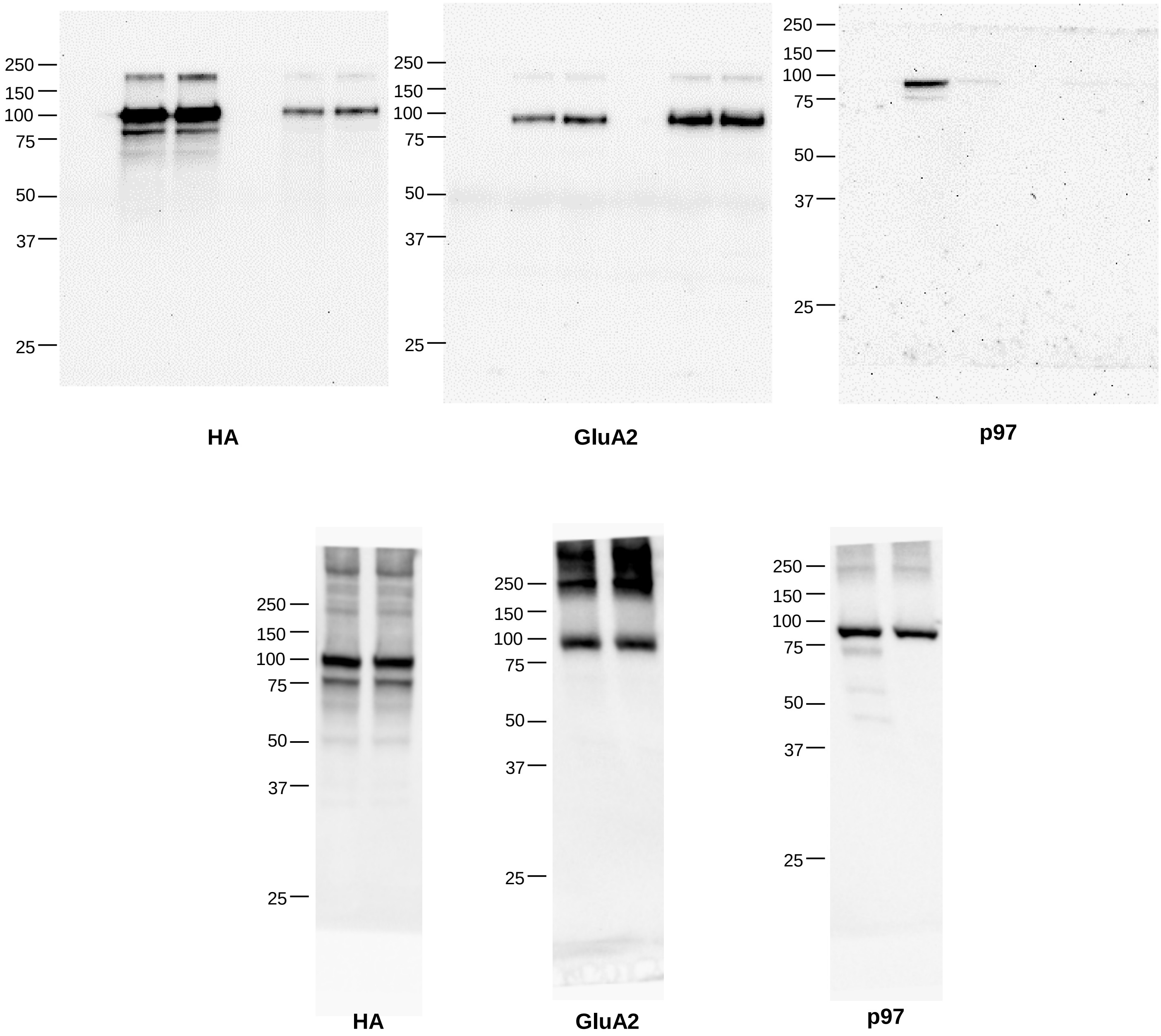

Fig 2e

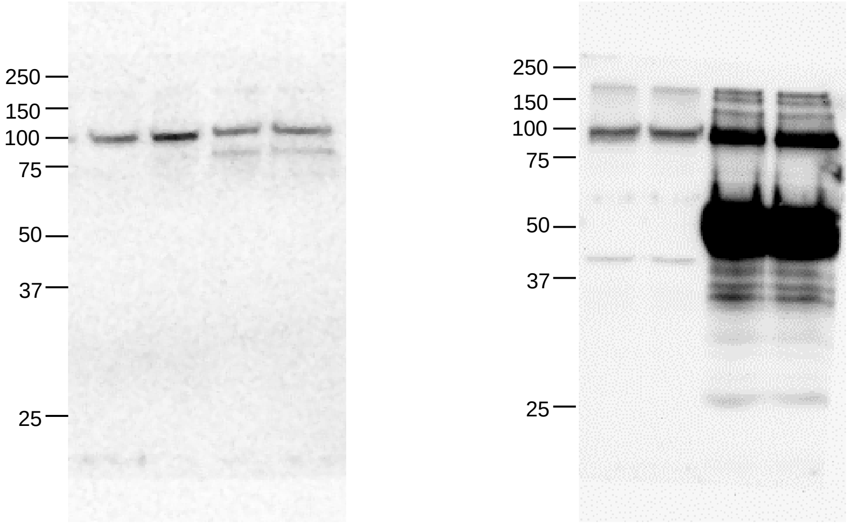

HA **GluA2**

Fig 3a

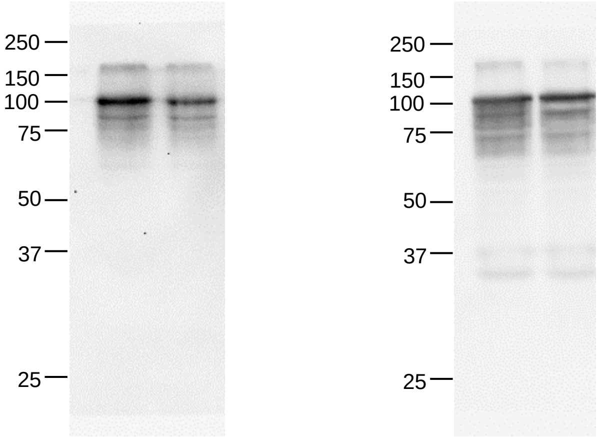

HA **HA**

Fig 5f

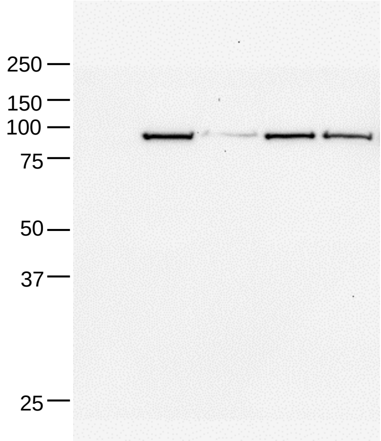

p97

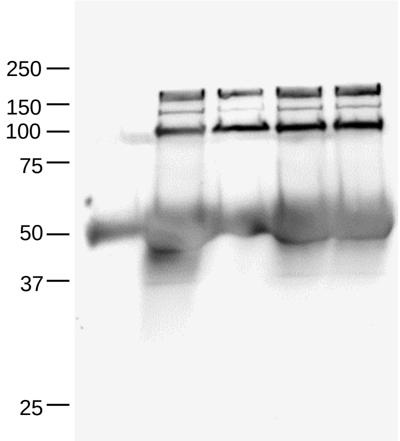

GluA1

Fig 5h

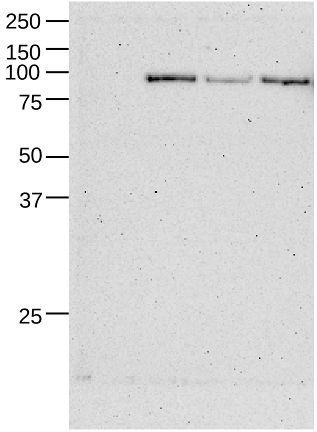

p97

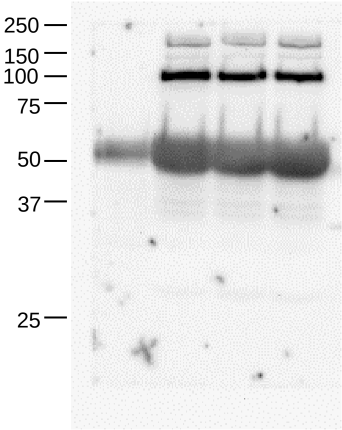

GluA1

Fig 6a

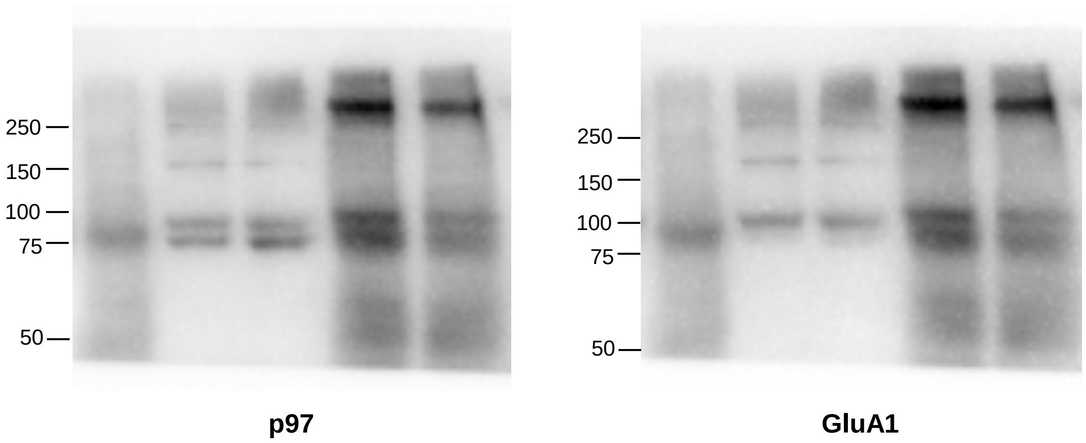
250
150
100
75
50
p97
250
150
100
75
50
GluA1

Fig 6c

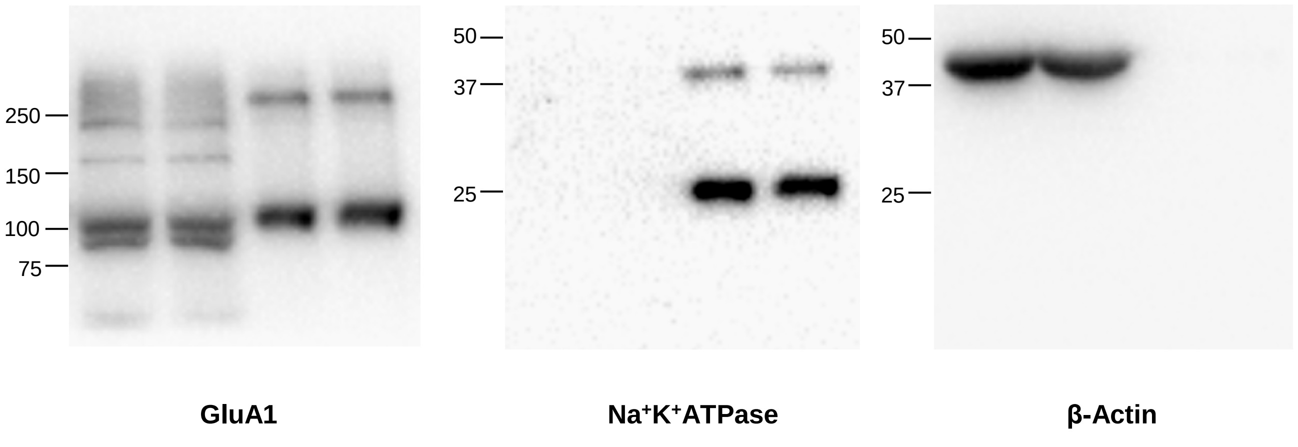
250
150
100
75
GluA1
50
37
25
Na+K+ATPase
50
37
25
β-Actin

Fig 6e

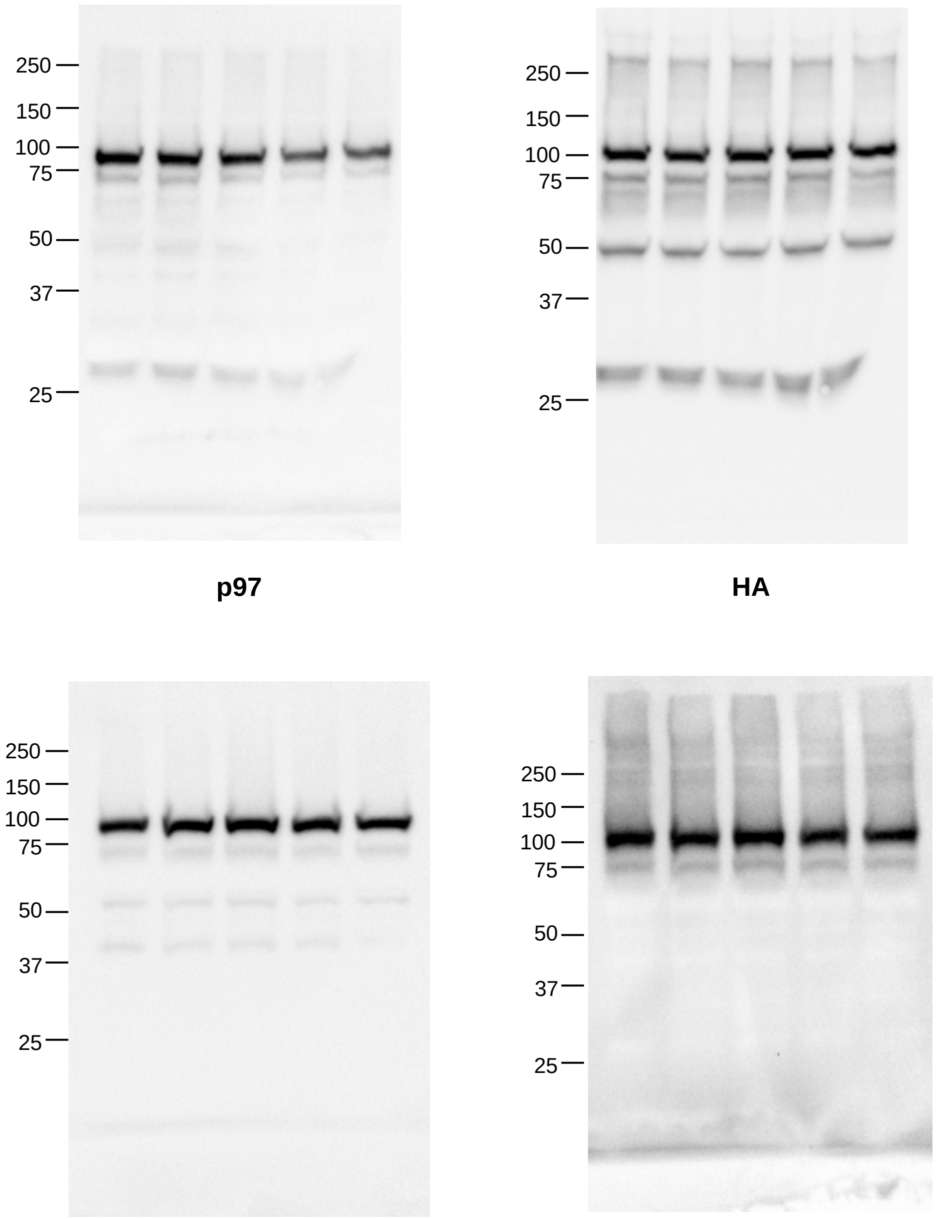

p97 **HA**

p97 **HA**
